# OnabotulinumtoxinA: Still the Present for Chronic Migraine

**DOI:** 10.3390/toxins15010059

**Published:** 2023-01-10

**Authors:** Carlo Baraldi, Flavia Lo Castro, Raffaele Ornello, Simona Sacco, Luca Pani, Simona Guerzoni

**Affiliations:** 1 Department of Biomedical, Metabolic and Neural Sciences, PhD School in Neurosciences, University of Modena and Reggio Emilia, 41124 Modena, Italy; 2 Department of Biomedical, Metabolic and Neural Sciences, Post Graduate School of Pharmacology and Clinical Toxicology, University of Modena and Reggio Emilia, 41124 Modena, Italy; 3 Department of Applied Clinical Sciences and Biotechnology, University of L’Aquila, 67100 L’Aquila, Italy; 4 Department of Biomedical, Metabolic and Neural Sciences, Pharmacology Unit, University of Modena and Reggio Emilia, 41124 Modena, Italy; 5 Department of Psychiatry and Behavioral Sciences, University of Miami, Miami, FL 33136, USA; 6 VeraSci, Durham, NC 27707, USA; 7 Department of Specialist Medicines, Digital and Predictive Medicine, Pharmacology and Clinical Metabolic Toxicology-Headache Center and Drug Abuse, Laboratory of Clinical Pharmacology and Pharmacogenomics, AOU Policlinico Di Modena, 41124 Modena, Italy

**Keywords:** OnabotulinumtoxinA, headache, pain, chronic migraine

## Abstract

OnabotulinumtoxinA (BT-A) is one of the few drugs approved for the preventive treatment of chronic migraine (CM). Despite this, some aspects of its mechanism of action are still a matter of debate, and the precise magnitude of BT-A effects needs to be completely elucidated. BT-A acts primarily upon trigeminal and cervical nerve endings, by inhibiting the release of inflammatory mediators such as calcitonin gene-related peptide, as well as reducing the insertion of ionotropic and metabotropic receptors into the neuronal membrane. These actions increase the depolarization threshold of trigeminal and cervical nerve fibers, thus reducing their activation. The central actions of BT-A are still a matter of debate: a retrograde axonal transport has been postulated, but not clearly assessed in humans. Clinically, the efficacy of BT-A in CM has been assessed by large, randomized placebo-controlled trials, such as the Phase 3 REsearch Evaluating Migraine Prophylaxis Therapy (PREEMPT) trials. Those results were also confirmed in a wide range of open-label studies, even for long-term periods. Recently, novel findings have led to a better understanding of its pharmacological actions and clinical usefulness in migraine prevention. This narrative review summarizes, updates and critically revises the available data on BT-A and its possible implementation in chronic migraine. Moreover, the current role of BT-A in CM treatment has been discussed.

## 1. Introduction

Chronic migraine (CM) is diagnosed when a patient experiences migraine attacks for ≥15 days per month, at least for 3 months [1]. CM affects around 1–2% of the worldwide population and is considered one of the main neurological disabilities, as it tremendously affects patients’ quality of life [2]. Moreover, patients with CM usually take large amounts of painkillers, which may paradoxically worsen CM itself, leading to a secondary headache called medication overuse headache (MOH) [3]. The burden of CM is further aggravated by the shortage of effective preventive treatments, which are often associated with poor efficacy and tolerability as early as the first months of treatment [4]. Moreover, the frequent association with MOH usually lowers the effectiveness of the preventive treatments, thus requiring painkiller withdrawal [5] before a preventive treatment could be started. Today, only topiramate, onabotulinumtoxinA (BT-A) and monoclonal antibodies targeting the calcitonin gene-related peptide (CGRP) or its receptor are specifically approved for the preventive treatment of CM [6]. Many randomized clinical trials (RCT), as well as real-life studies, pointed out the effectiveness of BT-A in CM treatment; however, some issues remain unresolved. Indeed, it is not clear where its pharmacological effect takes place and its exact magnitude. Moreover, BT-A indications should also be reconsidered, stating the availability of anti-CGRP drugs in the therapeutic armamentarium against CM. The aim of the present review is to discuss and critically reconsider the current pre-clinical and clinical data available on BT-A in CM.

## 2. Materials and Methods

A data search via Embase, MEDLINE, Web of Sciences, Google Scholar, and Clinical Trials.gov (30 September 2022) was performed, as suggested in previous work by Bramer et al. [7]. In particular, the following was conducted using the following non-MESH terms: “onabotulinumtoxinA” AND “chronic migraine”. Only articles published up to 30 September 2022 were considered. Originally, 507 articles were found. For each one, the full text was analyzed in order to decide its inclusion in the article. Additionally, reference lists of relevant original research and/or reviews were also reviewed to identify any clinical and/or preclinical investigations related to the purposes of this article. In particular, pre-clinical studies were considered only if referred to a trigeminal pain model. Only clinical studies adopting the Phase 3 REsearch Evaluating Migraine Prophylaxis Therapy (PREEMPT) injection paradigm (155 or 195 IUs in 31–38 pericranial sites) [8] on adult patients were included in this review. Real-life studies with fewer than 50 patients were not considered, in order to increase the reliability of the results [9]. Only English-written articles were considered. Abstracts and book chapters were excluded.

## 3. Current Understanding of Migraine Pathophysiology with Relevance for BT-A Treatment

### 3.1. Anatomy

The trigeminal nerve conveys pain signals from the anterior two-thirds of the scalp [10], whilst the posterior third is innervated by the second and third cervical nerves [11]. First-order trigeminal neurons are pseudo-unipolar and are located in the trigeminal ganglion (TG) [10]. The peripheral branches of their axons project peripherally to the meninges and cranial dermatomes, whereas the central projections synapse with second-order trigeminal neurons located in the brainstem into the trigeminal nucleus caudalis (TNC) [10,11]. In particular, the peripheral branches of first-order trigeminal neurons are unmyelinated C-fibers or poor myelinated Aδ-fibers which reach the meninges mainly with the ophthalmic branch of the V cranial nerve and, to a lesser extent, with the maxillary and mandibular ones. These fibers terminate freely in the dura mater, surrounding meningeal arteries or terminating, to a lesser extent, around veins, capillaries, or in poorly vascularized zones [12]. These fibers send two more branches, one reaching the pia mater [12] and the other one crossing the skull through cranial sutures to reach the periosteum of the skull, pericranial muscles and the skin [11]. The extensive discussion of the meningeal distribution of the trigeminal fibers is beyond the scope of this article, but it is summarized in a review by Levy and co-workers [12]. In a similar way, the occipital and supraclavicular nerves are formed by the peripheral branches of the axons of the pseudounipolar neurons located in the second and third cervical ganglion. These axons are C-fibers or Aδ-fibers that terminate peripherally in the skin, periosteum, and pericranial muscles located in the posterior third area of the scalp, sending collateral branches that cross the cranial sutures, bone canals and foramen magnum to reach the TNC, thus contributing to the trigeminal pain [13]. Therefore, the skull appears to be englobed by a wide network of intracranial and extracranial nociceptive fibers, originating from the V cervical nerve as well as from the second and third cervical nerves [14]. The pain signals conveyed by the trigeminal and the cervical fibers are integrated into the TNC, which can be considered as the “hub” of peripheral stimuli in migraine [15]. The axons of second-order neurons in the TNC decussate and reach the ventral posteromedial nucleus (VPMN) of the contralateral thalamus through the trigeminothalamic tract. The VPMN of the thalamus contains the third-order neurons that project to the primary and secondary somatosensory cortexes [10].

The abovementioned anatomic considerations are necessary to understand:

1. The injection protocol used for CM;
2. The ability of BT-A to inhibit cranial nociceptor activation toward both intracranial and extracranial stimuli.

### 3.2. Spotlights of Mechanisms in CM Justifying BT-A Use

#### 3.2.1. Stimuli Potentially Activating Cranial Nociceptive Fibers

Pain in CM arises from the activation of the nociceptive fibers innervating the meninges and the cranial dermatomes. These fibers may be activated by a wide range of stimuli (mechanical, thermal, chemical) because of the presence on their membranes of different kinds of receptors, such as the transient receptor potential vanilloid type 1 receptor (TRPV1); transient receptor potential ankyrin 1 receptor (TRPA1); and transient receptor potential membrane protein 8 (TRPM8) [16]. Moreover, the fact that both trigeminal and cervical fibers cross the skull gives the reason for their activation by intracranial as well as extracranial stimuli [17]. Among the first, cortical spreading depression (CSD) may activate nociceptive fibers [18]. Specifically, CSD is a cortical wave of neuronal and glial depolarization propagating at a speed of 2–6 mm per minute and followed by a long hyperpolarization (20–30 min). CSD activates meningeal nociceptors through the diffusion of small molecules such as nitric oxide (NO), potassium ions (K+), adenosine triphosphate (ATP) or hydrogen ions (H+) in the superficial cortical layer and their subsequent diffusion through the pia mater, arachnoid and dura mater [18]. The small molecules released by the abovementioned events act upon receptors such as the TRPV1, the TRPA1 and the TRPM8 [19]. Notably, as CSD is the physiological correlate of migraine with aura, this finding may justify the connection between aura symptoms and pain [20]. Extracranial stimuli activating nociceptive fibers may be physical, such as the mechanical deformation of the skin that can trigger the release of adenosine triphosphate (ATP) from keratinocytes and the activation of purinergic receptor P2X ligand-gated ion channel 3 (P2X3) expressed on the membrane of sensory nerve terminals [21]. Thermal stimuli may activate trigeminal nerve fibers as well, through the activation of TRMP8 channels [21]. Chemical stimuli, such as capsaicin, may activate TRPV1 channels [21]. The abovementioned receptors are ionotropic, and their exposure to a specific stimulus induces conformational changes and an increase in membrane conductivity towards cations, thus determining the depolarization of the cranial nociceptive fibers.

#### 3.2.2. Consequences of the Generation of an Action Potential in the Cranial Nociceptors

Regardless of the stimulus, if the depolarization of the neuronal membrane is supra-threshold, an action potential is generated and propagates both orthodromically and antidromically [22]. The antidromic conduction of the action potential increases the exocytosis of large dense-core vesicles from the termination of both C- and Aδ-fibers [23], whereas the orthodromic conduction determines the same phenomena at the level of the nerve bundles, which are not surrounded by Schwann’s cells. It ought to be taken into consideration that the abovementioned phenomena usually happen at a quantile level even in the basal conditions, but, if driven by an action potential, are more important. As a whole, the action potential causes the opening of the voltage-gated calcium channels, thus inducing the flow of Ca^2+^ inside the neurons [15] and the subsequent activation of the soluble N-ethymalemide-sensitive factor attachment protein receptor (SNARE) complex. The activation of the last complex is critical for the exocytosis processes. The exocytosis of large dense core vesicles has two main implications for migraine pathogenesis: the exocytosis of neurotransmitters and neuromodulators [24] as well as the insertion of receptors in the plasma membrane of cranial nociceptive fibers [25]. In particular, trigeminal nerve fibers contain large dense core vesicles containing CGRP, substance P, pituitary adenylyl cyclase-activating polypeptide 38 (PACAP-38) and receptors such as TRPV1, TRPA1 and P2X3 [21]. The exocytosis of neuromodulators determines the induction of a state of sterile neurogenic inflammation at the meningeal level, for which the most well-known actor is the CGRP [24]. For this reason, the discussion will take over on CGRP. The exocytosis of the CGRP determines three main phenomena. The first one is vasodilation at the meningeal level, as unequivocally demonstrated by an experiment with CGRP-blocking antibodies in rats [26]. Furthermore, the action of CGRP on pericytes at the endothelial levels determines an increase in the permeability of meningeal arteries [27]. Furthermore, the degranulation of meningeal mast cells has been linked to trigeminal nerve fiber activation [28]. It should be noticed that meningeal immune cells and CGRP are a growing field of research, but their relationship has not been completely unveiled yet. Despite this, readers may find an exhaustive review on this topic [28]. Moreover, CGRP also binds upon its receptors located on the Aδ-fibers, thus activating the PKA pathway and, consequently, trigeminal fiber sensitization [29]. On the other hand, the increase in the receptor expression upon the membrane of the cranial nerve fibers determines a reduction in the activation threshold of these fibers and, consequently, a higher probability of action potentials being generated [30].

#### 3.2.3. Central Transmission of the Pain Signals

After that, an action potential is transmitted towards the pyrenophore of the trigeminal and/or cervical neurons and through the centripetal branch of their axon, thus synapsing with the second-order neurons located in the TNC [10,22]. It should also be considered that the pain signal transmission at a cranial level is much more complex than described with the possibility of different neurons influencing the activity of the neighboring neurons, both at the level of the axon [31] and the pyrenophore [32]. At the central terminals, glutamate, serotonin and NO activate excitatory receptors on second-order neurons in the TNC in a frequency-dependent manner, which in turn displays an enhanced response following repeated stimuli [33]. Given these issues, in CM, trigeminal first-order neurons are sensitized at two levels: at the axon and at the pyrenophore [34]. Indeed, CM is often accompanied by an enhanced sensitivity to a light touch applied to the skin (tactile allodynia), reflecting the somatic convergence upon the same pool of second-order neurons receiving meningeal inputs [35]. Hence, CM may be considered as a referred pain mechanism, reflecting the convergence of sensory afferents originating from intracranial and extracranial structures to the second-order neurons in the TNC [35,36]. The distinction between intracranial and extracranial stimuli activating cranial nociceptors is clinically translated by the feature of an “imploding headache” as well as an “exploding headache”, respectively [37]. As BT-A is more effective in the relief of the imploding headache, it is more effective in inhibiting the activation of cranial nociceptors towards extracranial stimuli. Obviously, the present dissertation on migraine pathophysiology only considers the mechanism which can have relevance for BT-A treatment. Despite a growing amount of evidence pointing out the involvement of central mechanisms in CM pathophysiology, they will be only partially discussed.

## 4. Putative Mechanisms of BT-A in Migraine

**Figure 1:**
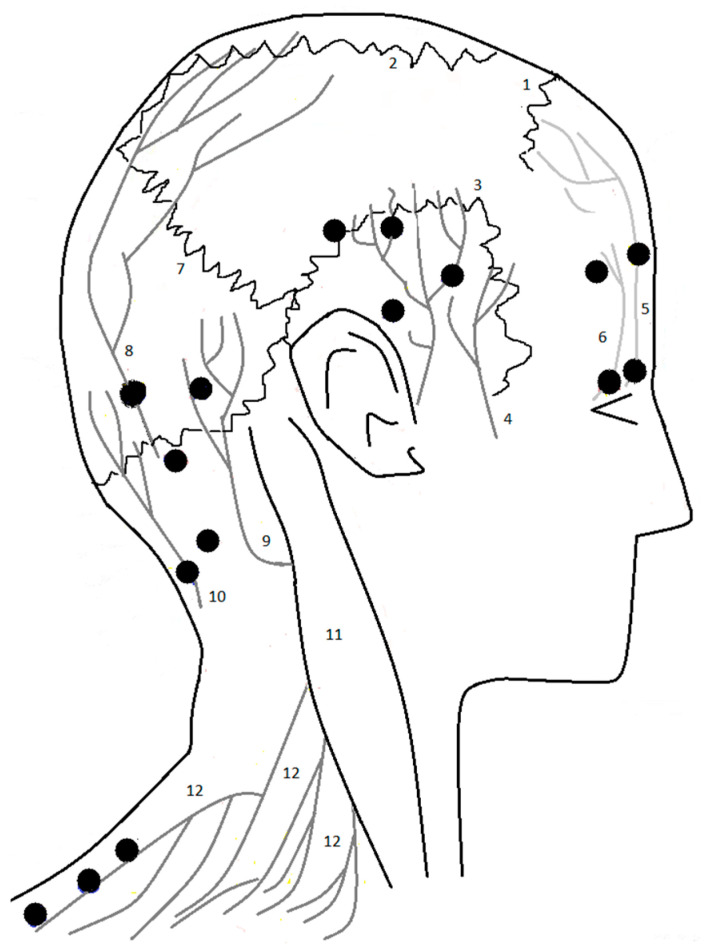
The points of the injections of BT-A and the nearby nerves from the PREEMPT 1 and 2 studies. Legend: 1: coronal suture; 2: sagittal suture; 3: squamosal suture; 4: auriculotemporal nerve; 5: supratrochlear nerve; 6: supraorbital nerve; 7: lambdoid suture; 8: greater occipital nerve; 9: lesser occipital nerve; 10: Third occipital nerve; 11: sternocleidomastoid muscle; 12: supraclavicular nerve.

The abovementioned mechanisms are fundamental to understanding the rationale behind the action of the BT-A in CM and, primarily, the rationale sustaining the internationally accepted injection protocol [8]. According to the PREEMPT protocol, the injections of the BT-A are given in 31 different points localized in muscles: frontalis, corrugator, procerus, temporalis, occipitalis, trapezius and cervical paraspinal muscle group. Additionally, up to 40 IU of BT-A may be administered using a “follow the pain” strategy into the temporalis, occipitalis or trapezius muscles [8]. Those areas correspond to the main nerves containing the peripheral branches of the axons of the trigeminal and cervical primary neurons (supraorbital, supratrochlear, zygomaticotemporal and auriculotemporal nerves) and in the second and third cervical ganglions (greater, lesser and accessory occipital nerves, supraclavicular nerves). These points are graphically summarized in Figure 1.

The possibility for extracranial-injected BT-A to influence the activity of intracranial neurons derives from the presence of intracranial nociceptive fibers that send collaterals crossing the skull through the sutures and emissary vein channels in mice, rats and humans [38,39,40]. Notably, a new version of the injection protocol specifically targets the sutures, i.e., the points in which the cranial nociceptive fibers enter the skull [41]. BT-A is a 900 kDa complex consisting of a 150 kDa botulinum neurotoxin associated with non-toxic proteins (neurotoxic-associated proteins—NAPs). The NAPs play a role in the pharmacological stability of the neurotoxin, determining its stability and protecting it from proteolysis. After the injection in the dermis, the NAPs rapidly dissociate from BT-A itself at a neutral pH, due to conformational changes, thus allowing BT-A to diffuse through extracellular space and reach the external projection of the trigeminal as well as the cervical neurons, which pass through the skull and reach the cranial dermatomes [42]. Probably, a still-unquantified, small amount of BT-A does not bind to the nerve fibers, and it is probably washed out from the lymphatic circulation [43]. The 150 kDa BT-A neurotoxin is composed of a light chain of 50 kDa (which contains the catalytic domain) and a heavy chain of 100 KDa. The last one is important for stability and transit, and the cellular penetration of the light chain is linked with it via a disulfide bond. The C-terminus of the heavy chain binds to the glycoproteins of the neuronal membrane surface, mainly trisialoganglioside GT1b and ganglioside GD1a [44]. The binding between GT1b and GD1a induces an initial, low-affinity binding to the neuronal membrane. After that, the interaction with the synaptic vesicle protein 2 (SV2) [45] or with the fibroblast growth factor receptor 3 (FGFR3) [46] induces clathrin-mediated endocytosis [47]. Interestingly, some evidence pointed out a role even for TRPV1 in this process [48]. Moreover, the N-terminus of the heavy chain (HN) may also be involved in the specific neuronal binding via interaction with phosphatidyl inositol phosphates at the presynaptic plasma membrane [49]. Once endocytosed, the BT-A mainly enters into the acidic vesicles, whereas the smaller fraction that enters non-acidic vesicular compartments may be sorted into the microtubule-dependent retrograde axonal transport pathways. The BT-A that enters the non-acidic compartment is sorted into the microtubule-dependent retrograde axonal transport towards the Gasser’s ganglion [49]. The entrance of the BT-A in the acidic vacuole determines its degradation into two parts: the 100 kDa remains in the acidic vacuole, whereas the 50 kDa domain is released into the cytoplasm and enters the cytosol through the reduction of the dysulfidrile bond that links the two parts [50]. The 50 kDa subunit concentrates near the inner layer of the plasma membrane thanks to its interactions with septins and the recruiting of specialized enzymes that inhibit ubiquitination [51,52]. This may explain the long-term persistence of BT-A action in nerve terminals, which is up to 1 year in cultured neurons and about 5 months in vivo [51]. The BT-A light chain is a Zn^2+^ dependent metalloprotease that cleaves the synaptosomal-associated protein of 25 kDa (SNAP-25), thus forming SNAP-25 (1–197), which is inactive and forms heterotrimers with other SNARE proteins to create inactive complexes [53]. The cleavage of SNAP-25 inhibits the exocytosis processes, thus limiting neuropeptides’ and neurotransmitters’ exocytosis and reducing the presentation of receptors on the plasma membrane [54]. Many pre-clinical papers have unveiled the mechanisms of action of BT-A in CM. Firstly, BT-A is able to inhibit the firing of meningeal nociceptors activated by cortical spreading depression in female rats, thus demonstrating the capability of BT-A to inhibit the activation of the meningeal nociceptor towards intracranial stimuli [55]. Among extracranial stimuli, BT-A has also been proven to inhibit the mechanical stimulation of the meningeal nociceptors, and, notably, BT-A inhibited only C-fibers and was also more effective in inhibiting the branch of peripheral nociceptors, but not the dural axon [56]. Moreover, a reduction in the response mediated by the TRPV1 and TRPA1 has also been detected in the peripheral branches of the cranial nociceptive neurons [57,58]. Interestingly, a decrease in the expression of TRPV1 was detected even at the level of the TG [25]. Furthermore, a decrease in the release of CGRP from the trigeminal neurons has been demonstrated, at the levels of both the peripheral branch of the axon [58] and the pyrenophore inside the TG [59]. Notably, a reduction in peripheral inflammation and peripheral sensitization has been found in human models of trigeminal sensitization [60,61]. It ought to be taken into consideration that cranial C-fibers contain the largest amount of CGRP, whereas Aδ-fibers have the highest expression of the CGRP receptor. Therefore, BT-A inhibits mainly C-fibers rather than Aδ ones [56]. Additionally, BT-A has been shown to undergo retroactive transport to influence the trigeminal neurons’ activity even at the level of TNC [62,63], as this effect is blocked by colchicine [64,65]. Despite this, not enough evidence is present to prove that BT-A may have central effects [66].

## 5. Clinical Use of BT-A in the Treatment of Chronic Migraine

### 5.1. Clinical Trials

#### 5.1.1. PREEMPT-1 and PREEMPT-2 Protocols

The first trials exploring the efficacy and safety of BT-A for the preventive treatment of CM were the PREEMPT ones. In particular, two randomized placebo-controlled trials were conducted. In the PREEMPT-1 trial, no significant differences were found between the BT-A and the placebo groups regarding the reduction in the number of headache episodes (−5.2 vs. −5.3; *p* = 0.344). Moreover, in the treated group, there was a significantly higher reduction in the number of headache days (*p* = 0.006), the number of migraine days (*p* = 0.002) and the number of triptans taken (*p* = 0.023) [67]. In the second PREEMPT-2 trial, 347 patients were randomly assigned to BT-A and 358 to placebo. In this trial, a significantly higher change from the baseline in the frequency of headache days (*p* < 0.001), in the frequency of migraine days (*p* < 0.001), in the frequency of moderate/severe headache days (*p* < 0.001) and in the HIT-6 score (*p* < 0.001) was found [68].

#### 5.1.2. Pooled Results of the PREEMPT-1 and PREEMPT-2 Protocols

A pooled analysis of the PREEMPT-1 and PREEMPT-2 results showed that BT-A (n = 688) determined a significant reduction in the number of headache days per month, the number of migraine days per month and the number of moderate/severe headache days per month after 24 weeks of treatment following the PREEMPT protocol [69]. A sub-group analysis by Lipton and coworkers performed on the pooled analysis unveiled that patients treated with BT-A experienced a significant amelioration in their quality of life, as witnessed by the 6-items headache impact test (HIT-6) and the Migraine-Specific Quality of Life Questionnaire v2.1 (MSQ) after 24 weeks of treatment [70]. Moreover, patients treated with BT-A also displayed bigger ameliorations of the HIT-6 score and of the MSQ score already after 12 weeks of treatment [70]. Furthermore, Silberstein and collaborators compared the effects of BT-A vs. placebo for the preventive treatment of CM in patients who displayed medication overuse at the baseline [71]. Interestingly, BT-A was superior to the placebo in the 24th week in terms of headache reduction and in the amelioration of the quality of life [71]. Among the painkillers, a significant decrease was seen for triptan and ergots, suggesting that BT-A is more effective on migraine-specific attacks rather than the ones with a tensive component [71].

#### 5.1.3. Pooled Results of the Open-Label Phase of the PREEMPT-1 and PREEMPT-2 Protocols

The PREEMPT trials foresaw a first double-blind 24-week-long phase, followed by a 32-week, open-label, single-treatment phase. Aurora’s group was the first to publish the results of even the open-label phase, still highlighting some significant differences between the patients taking BT-A or a placebo at the end of the observational period [72]. In particular, a significant reduction compared to the placebo was detected in terms of the frequency of headache and/or migraine days and in the number of moderate/severe headache days at 36 and 48 weeks. After the end of the open-label phase, the abovementioned parameters remained significant over the course of 1 year [72]. The ameliorations in the quality of life were even maintained after the open-label phase of the PREEMPT trial. Indeed, as Lipton and coworkers demonstrated, the HIT-6 and MSQ ameliorations observed in patients who received BT-A in the first 24-week-long phase were maintained even in the open-label phase of the trial, but not until the 56th week [73].

#### 5.1.4. Speed of Action of the BT-A from the PREEMPT-1 and PREEMPT-2 Protocols Pooled Analysis

The ameliorations in the quality of life found by Lipton and co-workers may also be attributed to a drastic reduction in the days with severe headache attacks, which was higher for the group treated with BT-A in the PREEMPT trial, whereas in the open-label phase, these differences were null [74]. In particular, the benefits of BT-A spread rapidly, as also witnessed by the percentage of patients experiencing a ≥50% response, which was about half of the patients after 12 weeks [75]. Despite the effectiveness that BT-A has demonstrated vs. placebo in treating MOH, BT-A did not afford any additional benefit over acute withdrawal alone in a recent trial conducted in patients with CM and MOH, neither on headache frequency nor on quality of life, disability or other outcome measures [76]. Despite this, another article highlighted how the effects of BT-A spread even after 1 week, thus suggesting a rapid onset of BT-A activity [77].

#### 5.1.5. BT-A vs. Topiramate

Additionally, BT-A was also explored in some RCTs against the only other drug specifically approved for the preventive treatment of CM, i.e., topiramate. The comparison between BT-A and topiramate was explored in the FORWARD study, demonstrating that the proportion of ≥50% responders was significantly higher in the group treated with BT-A [78,79]. RCTs regarding BT-A in CM are summarized in Table 1.

### 5.2. Real-Life Studies

Several real-world studies have been published, with findings consistent with PREEMPT studies in CM patients with and without MOH [80,81,82,83,84,85,86,87,88,89,90,91,92,93,94,95,96,97,98,99,100,101,102,103,104,105,106,107,108,109,110,111,112,113,114,115,116,117,118,119,120,121,122,123,124,125,126,127]. Real-world studies primarily exploring the effectiveness and safety of BT-A compared to the baseline are summarized in Table 2. One of the largest real-life studies was aimed at measuring healthcare resource utilization, and patient-reported outcomes observed in clinical practice (REPOSE study) [103,107]. In 633 patients with CM who received at least one dose of BT-A during a 2-year clinical routine, the CM frequency significantly diminished at all post-baseline visits, with improvements reported also in quality of life [103]. The results are congruent with a different group of 725 patients with CM, with the chances of a good outcome increased by starting treatment in the first 12 months after CM diagnosis [98]. Another big study was conducted by Khalil et al. on 254 adults with CM [80]. After one month of a single BT-A injection following the PREEMPT paradigm, there was a reduction in the number of headache and migraine days compared with the baseline, as well as an overall reduction in the number of days spent using analgesics and triptans [80]. Interestingly, the authors discovered that a longer CM duration and a higher disability as well as a higher consumption of painkillers at the baseline were negatively associated with a good response to BT-A after 1 year of treatment [80]. These results were substantially confirmed by Aicua-Rapun et al. [88]. B-TA has been demonstrated to be an effective and safe treatment even for long-time use. Indeed, different groups have unveiled that the BT-A effectiveness even lasts for 2 years of treatment [83,85,86]. Notably, some research groups have discovered that BT-A is effective even at longer time points, such as 3 years [95,99] and 4 years [108]. Apart from the prolonged effectiveness, it has been unveiled that the BT-A action spread fast, thus confirming the results of the PREEMPT study [76]. Indeed, many patients respond even at the first cycle [103,106]. Recently, a European collaboration has published different papers on a sample of 2879 patients in order to answer some still-open questions about the clinical use of the BT-A. Firstly, this group determined that two BT-A cycles are usually enough to establish if a patient is a responder or a non-responder to the BT-A. Indeed, patients who do not respond to the first two cycles of BT-A are unlikely to respond to the third cycle [125]. This indicates that, as BT-A effects spread rapidly, the first 6 months of treatment should be enough to establish if BT-A should be continued or not. Moreover, due to its relative expensiveness and the arrival of new anti-CGRP mAbs, it should also be important to identify patients who may benefit more from BT-A injections. In particular, the predictors of response towards BT-A are a higher CM duration, a higher disability and a higher VAS score at the baseline [98]. In the study by Dominguez and collaborators, no influence on MOH was detected. Despite this, Caronna et al. demonstrated the effectiveness of BT-A even in the treatment of CM complicated with MOH [102]. Negro et al. described the sustained effectiveness of the toxin for up to two years, even in patients complicated by MOH [85]. Specifically, BT-A reduced the number of migraine days and medication intake, also ameliorating HIT-6 scores [85]. Similar findings were described by Guerzoni and co-workers in a severely impaired population [86] and also for a longer time, up to 3 years [87]. Moreover, Ahmed et al. explored the effects of BT-A in 343 patients with CM, either overusing or not overusing medications [82]. Moreover, these data have been explored even up to 4 years, demonstrating stable effectiveness through time [88]. An improvement in the quality of life associated with a reduction in the HIT-6 score was also described [115,116,117,118]. Such findings were in accordance with another study conducted by Stark et al. [109]. In 211 patients with CM, around 74% of the treated patients achieved a relevant reduction in monthly headache days after only two treatment cycles, with concomitant lower use of acute headache medications [109]. BT-A’s efficacy has also been assessed on allodynia [110,111], which is frequent among chronic migraineurs and complicates their management. Young and co-workers clearly demonstrated that patients with and without allodynia similarly respond to BT-A [111]. Besides its effectiveness in difficult-to-treat patients, such as MOH sufferers and ones displaying allodynia, BT-A treatment response is also sustained in time. Indeed, about two-thirds of patients with CM achieving 50% or more response to BT-A within the third cycle of treatment maintain this positive response over time [110]. As CM is frequently associated with psychiatric comorbidities [2], different studies investigate the effects of BT-A on depressive symptoms [84,90,100]. Demiryurek et al. explored the activity of the toxin on depression and anxiety with the Beck Depression Inventory and the Beck Anxiety Inventory. After the third treatment cycle, only a slight improvement in the Beck Depression Inventory was achieved [90]. Similar results were obtained by Maasumi et al. using the nine-item Patient Health Questionnaire. In the latter study, a slight improvement in 359 patients was observed only at the third cycle [84]. A more recent study by Blumenfeld et al. demonstrated that the nine-item Patient Health Questionnaire and the seven-item generalized anxiety disorder score results were significantly lower compared to the baseline at all time-points until the ninth injection cycle [100]. By week 108, around 80% of the patients obtained a meaningful improvement in their depressive and anxiety symptoms [100]. Additionally, Aydinlar and collaborators found a significant reduction in the Migraine Disability Assessment (MIDAS) questionnaire score after the fifth BT-A injection compared to the baseline, but no improvements in the patients’ sleep quality [92]. Taken together, all this evidence points out also a favorable BT-A effect on the psychiatric comorbidities of CM, which may be an obstacle to its successful treatment, which is why it should be diagnosed early in CM. Gender does not influence patients’ response to BT-A [124]. Additionally, BT-A may also be a safe and valuable tool to treat CM in the elderly, when many treatments are not indicated [126].

### 5.3. Tolerability and Interactions

Adverse events (AEs) associated with BT-A are mainly local and transitory [80,81,82,83,84,85,86,87,88,89,90,91,92,93,94,95,96,97,98,99,100,101,102,103,104,105,106,107,108,109,110,111,112,113,114,115,116,117,118,119,120,121,122,123,124,125,126,127]. Muscular weakness, especially in the trapezius muscle, is one of the most common [116]. Other AEs include local itch, pain, inflammation, edema and swelling, mainly due to the mechanical stress related to the injection procedure. A flu-like syndrome and general malaise are rare but described. Both are possibly related to the systemic effects of BT-A. Post-injection headaches have also been reported; thus, BT-A injections should not be performed during migraine attacks. Such adverse events have been described for dosages of 155 U per treatment cycle, but also higher dosages have shown a similar safety profile [92]. Notably, BT-A safety is comparable to the placebo and maintained even after long-term therapies [87,96]. As a consequence, most patients were satisfied or extremely satisfied with BT-A treatment [88]. The overall incidence of adverse events and the most common individual events decreased with repeated BT-A administration [116]. In particular, the local AEs may be avoided by careful injection techniques, such as avoiding touching the periosteum or deep injections. Furthermore, as the extracranial prolongation of the trigeminal and cervical nerves cross the skull towards sutures and emissary canals, another way to reduce the potential of the BT-A inducing adverse events could be to practice injections only at the sutures [41]. Notably, some research groups have been focused on how ultrasound-guided injections improve the safety of BT-A injections. In particular, Kara et al. have described a new protocol for eco-guided injections of BT-A along the sutures, which improved the safety and effectiveness of the BT-A [128,129,130,131]. No interactions between BT-A and other drugs have been described so far.

### 5.4. Current Role of BT-A in the Treatment of Chronic Migraine

BT-A has the merit of being the first treatment specifically approved for the prevention of CM. The advent of BT-A shed light on a previously neglected disorder for which there were few available treatments with severe AEs and poor efficacy, which dramatically reduced patients’ compliance [4]. Beyond its effectiveness, the scheduling of the injections 3 months from each other increases patients’ compliance. Furthermore, BT-A has been demonstrated to have a more favorable cost-effectiveness profile than oral preventive anti-migraine drugs, especially in terms of a significantly lower likelihood of head-ache-related emergency department visits and hospitalizations [118]. Moreover, Rothrock and co-workers assessed that patients affected by CM experienced a significant cost offset during only the first 6 months of treatment [119]. The advent of BT-A also encouraged an interesting field of research in the pathophysiology of migraines, improving the understanding of the peripheral circuitry of migraine pain [43]. BT-A might also have central actions that are, however, yet to be defined on a mechanistic basis [62]. A large body of evidence from randomized and open-label studies summarized in this review supports the use of BT-A for the treatment of CM [67,68,69,70,71,72,73,74,75,76,77,78,79,80,81,82,83,84,85,86,87,88,89,90,91,92,93,94,95,96,97,98,99,100,101,102,103,104,105,106,107,108,109,110,111,112,113,114,115,116,117,118,119,120,121,122,123,124,125,126,127]. However, there are several open issues in the management of BT-A in clinical practice. For instance, it is unclear whether detoxication can enhance the preventive efficacy of BT-A in patients with medication overuse. BT-A is a proven treatment for medication overuse, in both randomized [71] and real-life [111] settings. However, a randomized trial emphasized the low value of detoxification treatments [76]. Moreover, good real-life data on the added value of combining detoxication and BT-A are lacking. Identifying predictors of response to BT-A could help avoid unnecessary treatments; however, the literature data in the field are inconclusive [99,132]. In the absence of clear selection criteria, a trial of BT-A can be offered to all patients with CM unless contraindicated. A further clinical issue is when declaring the failure of BT-A, as evidence suggests that patients not responding to the initial doses might respond later, even after one year [111], but, despite this, patients who do not respond to the first two cycles rarely found benefit from the third one [124]. A further clinical issue is whether treatment should be stopped in patients who present a sustained response to BT-A, as CM might undergo a rebound. An alternative approach to treatment stopping in those patients could be to increase the injection-free period from 3 to 4 or more months; however, the advantages of delayed injections have to be balanced with the potential wear-off of BT-A [105,121,122]. Moreover, some evidence suggests that BT-A suspension is associated with up to 6 months of well-being before CM relapses, at least in responders [106], but a recent article showed that the delay of BT-A due to the spread of the SARS-CoV-2 pandemic determines an overall worsening of migraines in patients previously affected by CM and MOH [123]. All these aspects of clinical practice are worth assessing in real-life studies. Moreover, BT-A could provide additional value when bruxism and/or temporomandibular joint disorder is present as a comorbidity to the CM [133]. Injecting additional BT-A into the masseter and temporalis muscles may not only ameliorate the bruxism but also improve the efficacy of PREEMPT. BT-A is now not the only specific preventive treatment for CM, as new treatments specifically targeting the calcitonin gene-related peptide (CGRP) or its receptor also represent a valid option [130]. In patients with CM that are resistant or have contraindications to several oral preventatives, it is uncertain whether BT-A or anti-CGRP mAbs should be used first. The choice between a monoclonal antibody against the CGRP or its receptor and BT-A should be targeted according to the patient’s headache history, comorbidities, and preferences. BT-A is particularly indicated in those patients who have an unfavorable cardiovascular risk profile or in older patients with polytherapy who might not tolerate treatments with a systemic action [127]. On the other hand, monoclonal antibodies might be preferred before BT-A in younger patients with a more favorable vascular risk profile. Notably, both BT-A and anti-CGRP mAbs act on the peripheral mechanisms of migraine pain. However, the peripheral action of BT-A is different from that of monoclonal Abs, paving the way for a possible combination of those two treatments in the future [134]. Indeed, the association between BT-A and anti-CGRP mAbs could be particularly indicated to reduce the wearing-off effect of BT-A, which is responsible for the reduction in the BT-A effect in the final part of the 3-month period between different toxin injections [107].

## 6. Conclusions

BT-A has been extensively studied in the preventive treatment of CM. Evidence that BT-A leads to a reduction in monthly headache and migraine days and improves quality of life originated from a series of clinical trials and real-world studies. Additionally, BT-A proved to be a safe medication, and patients are more comfortable than with traditional preventatives [85]. BT-A is also associated with lower health service utilization, especially for emergency departments [118]. In patients with CM and MOH, more work is necessary to clarify the usefulness of BT-A. Concerning its mechanism of action, there is still uncertainty about the true anti-nociceptive mechanism in CM, regarding in particular its hypothesized central effects.

## Figures and Tables

**Table 1:** Clinical trials of BT-A in migraine.

Study	Number of Patients	Time Point	Outcome	BT−A	Control	Mean Interchange Difference	*p*−Value
Aurora et al., 2010 (PREEMPT-1) [66]	679: 341: BT-A 338: Placebo	24 weeks	Change from baseline in the frequency of headache episodes	−5.2	−5.3	0.1 (−1.12, 0.39)	0.334
			Change from baseline in frequency of headache days	−7.8	−6.4	−1.4 (−2.40, −0.40)	0.006
			Change from baseline in frequency of migraine days	−7.6	−6.1	−1.5 (−2.60, −0.59)	0.002
			Change from baseline in frequency of migraine episodes	−4.8	−4.9	0.1 (−1.21, 0.26)	0.206
			Change from baseline in frequency of triptan intake	−3.3	−2.5	−0.8 (−1.69, −0.13	0.023
			Change from baseline of HIT-6 score	−4.7	−2.4	−2.3 (−3.25, −1.31)	<0.001
Diener et al., 2010 (PREEMPT-2) [67]	705: 347:BT-A 358: Placebo	24 weeks	Change from baseline in frequency of headache days	−9	−6.7	−2.3 (−3.25, −1.31)	<0.001
			Change from baseline in frequency of migraine days	−8.7	−6.3	−2.4 (−3.31, −1.36)	<0.001
			Change from baseline in frequency of moderate/severe headache days	−8.3	−5.8	−2.5 (−3.37, −1.48)	<0.001
			Change from baseline in cumulative total headache hours on headache days	−132.4	−90	−42.4 (−58.23, −21.05)	<0.001
			Percent of patients with severe (60) HIT-6 score	66.3	76.5	−10.2 (−16.9, −3.6)	0.003
			Change from baseline in frequency of headache episodes	−5.3	−4.6	−0.7 (−1.65, −0.33)	0.003
			Change from baseline in total HIT-6 scores	−4.9	−2.4	−2.5 (−3.54, −1.55)	<0.001
			Change from baseline in frequency of acute headache pain medication intakes (all categories)	−9.9	−8.4	−1.5 (−3.77, 0.49)	0.132
			Change from baseline in frequency of triptan intake	−3	−1.7	−1.3 (−2.24, −0.6)	<0.001
Dodick et al., 2010 (PREEMPT-1 and 2) [68]	1384 688: BT-A 696: Placebo	24 weeks	Change from baseline in frequency of headache days	−8.4	−6.6	−1.8 (−2.52, −1.13)	<0.001
			Change from baseline in frequency of migraine days	−8.2	−6.2	−2.0 (−2.67, −1.27)	<0.001
			Change from baseline in frequency of moderate/severe headache days	−7.7	−5.8	−1.9 (−2.62, −1.26)	<0.001
			Change from baseline in cumulative total headache hours on headache days	−119.7	−80.5	−39.2 (−48.40, −21.04)	<0.001
			Percent of patients with severe (≥60) HIT-6 score	67.6%	78.2%	−10.6% (−15.2%, −5.9%)	<0.001
			Change from baseline in frequency of headache episodes	−5.2	−4.9	−0.3 (−1.17, −0.17)	0.009
			Change from baseline in frequency of migraine episodes	−4.9	−4.5	−0.4 (−1.20, −0.23)	0.004
			Change from baseline in frequency of acute headache pain medication intakes (all categories)	−10.1	−9.4	−0.7 (−2.68, 0.69)	0.247
			Change from baseline in frequency of triptan intake	−3.2	−2.1	−1.1 (−1.74, −0.61)	<0.001
			Change from baseline in total HIT-6 scores	−4.8	.2.4	−2.4 (−3.11, −1.72)	<0.001
			Change from baseline in MSQ score				
			Role function−restrictive	17	8.6	8.4 (10.76, 6.01)	<0.001
			Role function−preventative	13.1	6.4	6.7 (9.01, 4.35)	<0.001
			Emotional function	17.9	9.5	8.4 (11.37, 5.56)	<0.001
Lipton et al., 2011 (PREEMPT-1 and 2) [69]	1384 688: BT-A 696: Placebo	12 weeks	Change from the baseline in the HIT-6 score	−4.7	−2.6	2.1	<0.001
			Change from the baseline in the MSQ score (restrictive)	16.2	9.9	6.3	<0.001
			Change from the baseline in the MSQ score (preventive)	13	8	5.0	<0.001
			Change from the baseline in the MSQ score (functioning)	18.3	11	7.3	<0.001
		24 weeks	Change from the baseline in the HIT-6 score	−4.8	−2.4	2.4	<0.001
			Change from the baseline in the MSQ score (restrictive)	17	8.6	8.4	<0.001
			Change from the baseline in the MSQ score (preventive)	13.1	6.4	6.7	<0.001
			Change from the baseline in the MSQ score (functioning)	17.9	9.5	8.4	<0.001
Silberstein et al., 2013 (PREEMPT-1 and 2) [70]	904 445: BT-A 459: Placebo	24 weeks	Change from the baseline in the frequency of headache days	−8.2 (0.3)	−6.2 (0.31)	−	<0.001
			Change from the baseline in the frequency of migraine	−8.1 (0.3)	−6 (0.31)	−	<0.001
			Change from the baseline in the frequency of moderate/severe headache days	−7.7 (0.29)	−5.7 (0.31)	−	<0.001
			Change from the baseline in the total cumulative hours of headache on headache days	−114.5 (5.77)	−70.8 (6.08)	−	<0.001
			Percent of patients with severe (≥60) HIT-6 score	71%	81.9%	−	<0.001
			Change from the baseline in the frequency of headache episodes	−5.4 (0.26)	−5.1 (0.25)	−	0.028
			Change from the baseline in the frequency of migraine episodes	−5.1 (0.25)	−4.8 (0.25)	−	0.018
			Change from the baseline in the frequency of acute headache medication intakes	−13.1 (0.9)	−11.8 (0.89)	−	0.21
			Change from the baseline in the total HIT-6 score	−4.7	−2.2	−	<0.001
			MSQ score (restrictive)	16.9	7.6	−	<0.001
			MSQ score (preventive)	13.9	5.8	−	<0.001
			MSQ score (functioning)	18.3	8.7	−	<0.001
			Change from the baseline in the frequency of triptan intake	−3.3 (0.22)	−2.4 (0.1)	−	<0.001
Aurora et al., 2011 (PREEMPT-1 and 2) [71]	1384 688: BoNT/A 696: Placebo	56 weeks	Change from baseline in mean frequency of headache days	−11.7 (−12.17, −11.20)	−10.8 (−11.32, −10.31)	−0.9 (−1.53, −0.14)	0.019
			Change from baseline in mean frequency of migraine days	−11.2 (−11.71, −10.74)	−10.3 (−10.82, −9.80)	−0.9 (−1.52, −0.14) 0.018	0.018
			Change from baseline in mean frequency of moderate/severe headache day	−10.7 (−11.18, −10.25)	−9.9 (−10.43, −9.44)	−0.8 (−1.41, −0.09) 0.027	0.027
			Change from baseline in cumulative total headache hours on headache days	−169.1 (−179.30, −158.81)	−145.7 (−155.94, −135.36)	−23.4 (−29.15, −2.78) 0.018	0.018
			Percent of patients with severe (60) HIT-6 score	50.6% (46.9%, 54.3%)	51.9% (48.2%, 55.6%)	−1.3% (−6.6%, 4.0%)	0.632
			Change from baseline in mean frequency of headache episodes	−7.4 (−7.79, −6.97)	−7.5 (−7.91, −7.09)	0.1 (−0.87, −0.04)	0.075
			Change from baseline in mean frequency of migraine episodes	−6.8 (−7.21, −6.43)	−7.0 (−7.37, −6.58)	0.2 (−0.80, −0.09)	0.117
			Change from baseline in mean frequency of acute headache medication intakes	−15.4 (−16.74, −14.05)	−15.7 (−17.05, −14.33)	0.3 (−1.76, −1.29)	0.76
			Change from baseline in mean frequency of triptan intakes	−4.2 (−4.69, −3.67)−3.8 (−4.35, −3.27)	−3.8 (−4.35, −3.27)	−0.4 (−1.02, −0.06) 0.080	0.08
			Change from baseline in mean frequency of acute headache medication days	−8.4 (−9.08, −7.79)	−8.5 (−9.16, −7.82)	0.1 (−1.19, 0.46)	0.387
			Change from baseline in total HIT-6 scores	−7.7 (−8.24, −7.06)	−7.0 (−7.62, −6.40)	−0.6 (−1.49, 0.20)	0.069
			Change from the baseline in the total MSQ score				
			MSQ score (restrictive)	25.2 (27.27, 23.08)	21.8 (23.93, 19.63)	3.4 (6.41, 0.39)	0.043
			MSQ score (preventive)	19.0 (21.06, 17.01)	17.3 (19.40, 15.26)	1.7 (4.60, 1.20)	0.293
			MSQ score (functioning)	25.0 (27.41, 22.60)	22.1 (24.66, 19.62)	2.9 (6.36, −0.62)	0.51
Lipton et al., 2016 (PREEMPT-1 and 2) [72]	1384 688: BoNT/A 696: Placebo	36 weeks	Change from baseline in total HIT-6 scores	−7	−5.8	−	0.002
			Percentage of patients with severe or substantial impact	68%	74%	−	0.022
			Percentage of patients with a ≥5 points decrease in the HIT-6 score	57%	51%	−	0.002
			Patients with a ≥50% decrease in the headache days	56%	53%	−	
			Percentage of patients with ≥5–point decrease in HIT-6 score and ≥50% reduction in headache days	40%	35%	−	0.022
		48 weeks	Change from baseline in total HIT-6 scores	−7.1	−6.1	−	0.022
			Percentage of patients with severe or substantial impact	68%	71%	−	>0.05
			Percentage of patients with a ≥5 points decrease in the HIT-6 score	56%	52%	−	>0.05
			Patients with a ≥50% decrease in the headache days	61%	57%	−	>0.05
			Percentage of patients with ≥5–point decrease in HIT-6 score and ≥50% reduction in headache days	38%	43%	−	>0.05
		56 weeks	Change from baseline in total HIT-6 scores	−7.7	−7	−	>0.05
			Percentage of patients with severe or substantial impact	68%	66%	−	>0.05
			Percentage of patients with a ≥5 points decrease in the HIT-6 score	59%	57%	−	>0.05
			Patients with a ≥50% decrease in the headache days	67%	61%	−	0.022
			Percentage of patients with ≥5–point decrease in HIT-6 score and ≥50% reduction in headache days	49%	43%	−	0.022
Mataharu et al., 2017 [73]	1384 688: BoNT/A 696: Placebo	24 weeks	Percentage of reduction in the severe headache days	41.1%	31.1%	−	0.011
		56 weeks	Percentage of reduction in the severe headache days	64.6%	65.6%	−	0.792
Silberstein et al., 2014 (PREEMPT-1 and 2) [74]	1384 688: BoNT/A 696: Placebo	12 weeks	Percentage of patients with a ≥50% reduction in the frequency of headache days	49.3%	−	−	−
			Percentage of patients with a ≥50% reduction in the frequency of moderate/severe days	53.1%	−	−	−
			Percentage of patients with a ≥50% reduction in the cumulative hours of headache on headache days	54.2%	−	−	−
			Percentage of patients with a ≥5 points reduction in the HIT-6 score	57.6%	−	−	−
Pijpers et al., 2019 [75]	179 90: BT-A 89: placebo	12 weeks	Change from baseline in mean frequency of headache days	−5.6	−4.4	−	0.7
			Change from baseline in mean frequency of migraine days	−6.2	−7	−	0.38
			Change from baseline in moderate/severe headache days	−4.9	−5.4	−	0.55
			Change in hours of headache	−20.8	−13.3	−	0.66
			Transformation of chronic migraine to episodic migraine	62.5%	57%	−	0.29
			25% responder rate	48.3%	37.8%	−	0.16
			50% responder rate	18.1%	20.4%	−	0.69
			Succeed to withdraw from medication	89.7%	89.8%	−	0.89
			Change from the baseline in the HIT-6 score	−0.8	−0.8	−	0.96
			Change from the baseline in the MIDAS score	18.7	24	−	0.67
Dodick et al., 2019 (PREEMPT-1 and 2) [76]	1384 688: BT-A 696: Placebo	1 week	Change from the baseline in the number of headache days per week	−0.9 ± 2.2	−0.7 ± 2.1	−	0.046
		3 week	Change from the baseline in the number of headache days per week	−1.6 ± 2.2	−1.1 ± 2.2	−	<0.001
		4 week	Change from the baseline in the number of headache days per week	−1.6 ± 2.2	−1.2 ± 2.2	−	<0.001
Aurora et al., 2014 (PREEMPT-1 and 2) [77]	1384 688: BT-A 696: Placebo	24 weeks	Change from the baseline in the frequency of headache days	−8.8 (−9.4, −8.2)	−6.5 (−7.1, −5.9)	−	<0.001
			Change from the baseline in the frequency of migraine days	−8.6 (−9.2, −8.0)	−6.2 (−6.7, −5.5)	−	<0.001
			Change from baseline in moderate/severe headache days	−8.2 (−8.7, −7.6)	−5.8 (−6.4, −5.2)	−	<0.001
			Change from the baseline in the cumulative headache hours on headache days	−121.8 (−135.9, −112.2)	−82.0 (−91.9, −67.3)	−	<0.001
			Change from the baseline in the frequency of headache episodes	−5.9 (−6.1, −5.2)	−4.8 (−5.4, −4.4)	−	<0.001
			Change from the baseline in the frequency of migraine episodes	−5.5 (−5.8, −4.9)	−4.4 (−5.0, −4.1)	−	<0.001
			Change in the frequency of medication intake for headache	−10.4 (−11.8, −8.7)	−9.3 (−11.0, −8.0)	−	0.293
			Change from the baseline in the frequency of triptan intake	−3.4 (−3.8, −2.8)	−2.1 (−2.8, −1.6)	−	<0.001
		56 weeks	Change from the baseline in the frequency of headache days	−12.0 (−12.6, −11.5)	−11.1 (−11.8, −10.5)	−	0.035
			Change from the baseline in the frequency of migraine days	−11.6 (−12.2, −11.0)	−10.7 (−11.3, −10.0)	−	0.038
			Change from baseline in moderate/severe headache days	−11.0 (−11.5, −10.4)	−10.1 (−10.7, −9.5)	−	0.042
			Change from the baseline in the cumulative headache hours on headache days 166.8 (182.7, 158.2)	−166.8 (−182.7, −158.2)	−151.2 (−160.5, −134.3)	−	0.063
			Change from the baseline in the frequency of headache episodes	−8.1 (−8.3, −7.4)	−7.5 (−8.3, −7.3)	−	0.057
			Change from the baseline in the frequency of migraine episodes	−7.5 (−7.7, −6.8)	−7.0 (−7.8, −6.8)	−	0.088
			Change in the frequency of medication intake for headache	−16.1 (−17.4, −14.1)	−16.1 (−18.2, −14.8)	−	0.939
			Change from the baseline in the frequency of triptan intake	−4.6 (−5.1, −3.9)	−4.2 (−5.0, −3.7)	−	0.166
Rothrock et al., 2019 [78]	282 140: BT−A 142: topiramate 50−100 mg	32 weeks	Percentage of ≥50% responders	40%	12%	4.9 [95% CI, 2.7 ÷ 9.1]	0.015
Blumenfeld et al., 2020 [79]	282 140: BT−A 142: topiramate 50−100 mg	32 weeks	Mean decrease in the HIT−6 score compared to the baseline	−	−	–4.25 [95% CI: –5.77, –2.73]	<0.001
			Mean total score of the PHQ−9	−	−	–1.86 [95% CI: –2.63, –1.10];	<0.001

**Table 2:** Real-life studies.

Study	Number of Patients	Length	Efficacy	Baseline	Last Time-Point	*p*-Value
Khalil et al., 2014 [80]	254	Variable	Headache days per month	27 (22,30)	18 (10,25)	<0.001
			Migraine days per month	15 (10,19)	7 (3,12)	<0.001
			Crystal clear days per month	3 (0,8)	12 (5,20)	<0.001
			Mild headache days per month	10 (7,15)	8 (4,13)	<0.001
			Days with painkillers per month	12 (7,20)	6 (2,12)	<0.001
			Days with triptans per month	5 (0,8)	2 (0,6)	<0.001
			Days off work per month	4 (3,6)	1 (0,4)	<0.001
Pedraza et al., 2015 [81]	52	12 weeks	Headache days per month	23.4 ± 6.3	12.8 ± 9.6	<0.001
			Migraine days per month	13.9 ± 7.3	5.3 ± 5.5	<0.001
			Days with painkillers per month	17.7 ± 9.2	8.7 ± 8	<0.001
			Days with triptans per month	5.1 ± 6.9	2.1 ± 3.6	<0.001
Ahmed et al., 2015 [82]	215 without medication overuse	12 weeks	Headache days per month	26 (20,30)	17 (11,28)	<0.001
			Migraine days per month	14 (10,20)	8 (4,12)	<0.001
			Crystal clear days per month	4 (0,10)	13 (3,19)	<0.001
			Days with painkillers per month	8 (2,10)	4 (0,8)	<0.001
			Days with triptans per month	2 (0,5)	0 (0,4)	<0.001
			Days off work per month	3 (3,5)	1 (0,3)	<0.001
	215 without medication overuse		Headache days per month	28 (24,30)	20 (12,26)	<0.001
			Migraine days per month	16 (12,20)	9 (5,15)	<0.001
			Crystal clear days per month	2 (0,6)	10 (4,18)	<0.001
			Days with painkillers per month	20 (16,28)	10 (5,18)	<0.001
			Days with triptans per month	6 (3,12)	2 (0,7)	<0.001
			Days off work per month	4 (2,8)	2 (0,4)	<0.001
Cernuda-Morollon et al., 2015 [83]	132	12 months	Responders		74.2%	
Maasumi et al., 2015 [84]	359	6 months	Patients with a ≥6 points decrease in the HIT-6 score		108 (30.1%)	
Negro et al., 2015 [85]	132	24 months	Headache days per month	22.3 ± 4.1	7.3 ± 2.1	<0.001
			Migraine days per month	21.4 ± 4.3	6.8 ± 2.3	<0.001
			Days with painkillers per month	20.8 ± 4.5	5.3 ± 1.7	<0.001
			HIT-6 score	69.4 ± 4.9	52 ± 5.6	<0.001
			Patients with severe (≥60) HIT-6 score	93.9%	22%	<0.0001
Guerzoni et al. 2015 [86]	57	24 months	Headache index	0.98 ± 0.09	0.65 ± 0.36	<0.0001
			Analgesic consumption	1.79 ± 1.59	0.61 ± 0.42	<0.0001
			Visual analogue scale for pain (VAS) score	7.98 ± 1.26	4.25 ± 1.48	<0.001
			HIT-6 score	63.94 ± 6.91	52.28 ± 8.69	<0.001
Vikelis et al., 2016 [87]	119	9 months	Headache days per month	21.3 ± 5.4	7.7 ± 4.8	<0.001
			Peak headache days per month	11.9 ± 5.5	3.7 ± 3.3	<0.001
			Days with a VAS > 4 per month	4–30	0–18	<0.001
			Days with any acute headache medication per month	16.2 ± 7.8	5.2 ± 4.3	<0.001
Aicua-Rapun et al., 2016 [88]	115	12 weeks	Patients remitting from CM to episodic migraine	68.7%	-	-
			Patients stopping other preventive treatments for migraine	42.5%	-	-
			Patients discontinuing MOH	61.9%	-	-
Russo et al., 2016 [89]	52	6 months	Headache days per month	20 (15,30)	18 (10,30)	0.002
			Days with painkillers per month	17.5 (15,28.8)	15 (9.3,28)	0.016
			Painkillers per month	20 (15,41.8)	15 (7,31)	0.014
		9 months	Headache days	19 (15,26.3)	14.5 (10,25.8)	0.011
			Days with painkillers per month	15 (15,25)	9 (5.5,17.5)	0.015
			Painkillers per month	20 (15,41.3)	12 (7.5,24)	0.005
Demiryurek et al., 2015 [90]	124	4 weeks	Headache days per month	18.78 ± 2.06	5.80 ± 4.17	
			Admissions to the emergency department	2.72 ± 1.28	0.47 ± 0.89	<0.001
			Painkillers per month	2.35 ± 0.88	0.67 ± 0.57	<0.001
			VAS score	8.90 ± 0.75	3.80 ± 2.17	<0.001
			Duration of attacks	2.63 ± 0.66	0.96 ± 0.66	<0.001
			Frequency of attacks	5.05 ± 1.20	1.55 ± 1.48	<0.001
		12 weeks	Headache days per month	18.78 ± 2.06	12.38 ± 3.98	<0.001
			Admissions to the emergency department	2.72 ± 1.28	1.27 ± 1.06	<0.001
			Painkillers per month	2.35 ± 0.88	1.18 ± 0.56	<0.001
			VAS score	8.90 ± 0.75	6.53 ± 1.44	<0.001
			Duration of attacks	2.63 ± 0.66	1.90 ± 0.68	<0.001
			Frequency of attacks	5.05 ± 1.20	3.37 ± 1.38	<0.001
			MIDAS score	17.40 ± 4.92	8.22 ± 5.29	<0.001
Negro et al., 2015 [91]	172	24 months	Headache days per month	22.2 ± 4.9	4.1 ± 1.0	<0.05
			Migraine days per month	21.6 ± 4.8	3.8 ± 1.0	<0.05
			Painkillers per month	21.0 ± 5.1	3.7 ± 1.3	<0.05
			HIT-6 score	67.9 ± 4.2	49 ± 6.7	<0.05
Aydinlar et al., 2017 [92]	190	12 months	Headache days	15.0 (12.0–25.0)	5.0 (2.0–10.0)	0.017
			VAS score	8.0 (7.0–9.0)	7.0(5.0–7.0)	0.023
			Painkillers per month	20.0 (15.0–30.0)	5.5(2.0–10.0)	<0.001
			MIDAS score	57.0 (35.5–75.0)	10.0(2.0–15.0)	0.002
			DASS-21 depression	85	7	0.002
			DASS-21 anxiety	85	7	0.002
			DASS-21 stress	85	7	0.002
			PSIQ	9.0 (5.0–12.0)	4.0 (1.0–7.0)	0.002
Matharu et al., 2017 [93]	1160	15 months				
Byun J et al., 2017 [94]	100	4 weeks	Good responders	12.6%	-	-
Guerzoni et al., 2017 [95]	90	36 months	Headache days per month	0.98 ± 0.16	0.49 ± 0.29	<0.001
			VAS score	7.66 ± 1.56	3.31 ± 1.25	<0.001
			Painkillers per month	1.98 ± 1.69	0.49 ± 0.29	<0.001
			HIT-6 score	65.1 ± 6.24	57.15 ± 5.7	<0.001
Dikmen et al., 2018 [96]	144	3 months	Headache days per month	18.80 ± 5.53	5.77 ± 5.06	0.001
			MIDAS-score	53.62 ± 24.84	16.17 ± 16.91	0.001
Blumenfeld et al., 2018 [97]	716	108 weeks	Headache days per month	22.0 [4.8]	11.3 ± 7.4	<0.0001
			Patients with a ≥50% reduction in headache days	-	61.1%	-
Dominguez et al., 2018 [98]	725	12 months	Headache days per month	21.8 ± 6.4	8.4 ± 5.7	<0.01
			Migraine days per month	13.8 ± 7.0	6.0 ± 4.7	<0.01
			Painkillers per month	17.0 ± 9.9	6.3 ± 8.3	<0.01
			Triptans per month	9.3 ± 8.7	4.8 ± 4.0	<0.01
			Admissions to the ED for headache	2.2 ± 2.6	0.9 ± 1.8	<0.01
			VAS score	7.6 ± 1.1	4.6 ± 2.1	<0.01
			MIDAS score	35.9 ± 29.6	9.1 ± 6.6	<0.01
			Responders	-	79.3%	-
Vikelis et al., 2018 [99]	56	36 months	Headache days per month	21.5 ± 5.1	3.4 ± 1.7	<0.001
			Days with moderate/severe headache per month	11.7 ± 5.7	2.5 ± 1.1	0.052
			Days with painkiller per month	16.5 ± 7.3	2.8 ± 1.3	<0.001
Blumenfeld et al., 2018 [100]	715	108 weeks	Headache days per month		−10.6 ± 7.4	<0.001
			Change in the PHQ-9 scores		−4.5	<0.001
			Change in the GAD-7 scores		−2.8	<0.001
Andreou et al., 2018 [101]	200	108 weeks	Headache days per month	23 (17,30)	8 (5,11.3)	<0.001
			Migraine days per month	13 (9,18)	4 (0,8)	<0.001
			Days with painkillers	10 (4,16)	3 (3.3,18.8)	<0.001
			Headache-free days per month	0 (0,5)	20.3 (10.7,22)	<0.001
			HIT-6 score	70 (66,72)	62 (56.5,66)	<0.001
Caronna et al., 2018 [102]	139	12 weeks	Headache frequency per month	27.3 ± 4.7	15.4 ± 9.9	<0.001
			Patients with daily headache	71.2%	23.2%	<0.001
			Migraine days per month	13.4 ± 8.1	6.5 ± 5.7	<0.001
			Headache days per month	13.8 ± 9.0	8.9 ± 8.1	<0.001
			Patients with daily painkiller intake	66.2%	13.7%	<0.001
Ahmed et al., 2019 [103]	633	21 months	Change of the headache days per month		−13.1	<0.001
			Change of the MSQ restrictive		33.6	<0.001
			Change of the MSQ preventive		28.9	<0.001
			Change of the MSQ emotional		34.9	<0.001
			Change of the EQ-5D score		0.2	<0.001
Quitas et al., 2019 [104]	193	24 weeks	Patients displaying a wear-off phenomenon	-	23.3%	-
Ching et al., 2019 [105]	131	24 weeks	Patients referring a worsening after BT-A stoppage	-	20%	-
Alpuente et al., 2019 [106]	105	108 weeks	Headache days per month	13.6 ± 8.2	8.5 ± 8.3	<0.001
			Migraine days per month	11.0 ± 6.5	5.2 ± 4.7	<0.001
			Headache frequency	24.4 ± 7.1	13.9 ± 10.0	<0.001
			MIDAS score	84.1 ± 79.7	54.1 ± 53.9	<0.001
			Oral preventive drugs	2.3 ± 1.1	1.3 ± 0.9	<0.001
Santoro et al., 2020 [108]	109	208 weeks	Headache days per month	25.5 ± 5.8	6.3 ± 3.3	<0.001
			Migraine hours per month	538.6 ± 176.1	36.4 ± 29.0	<0.001
Stark et al., 2019 [109]	211	24 weeks	Patients with a ≥50% response		74% (68–80%)	
			Reduction in the migraine days per month		− 9.4 ± 7.6	
			Reduction in the HIT-6 score		− 11.8 (12.2)	
Ornello et al., 2020 [112]	115	60 weeks	Headache days	30 (25–30)	15 (7–25)	<0.001
			Migraine days	30 (25–30)	15 (7–25)	<0.001
			NRS score	8 (7–9)	5 (4–7)	<0.001
			HIT-6 score	65 (60–69)	62 (56–65)	<0.001
			MIDAS score	87.5 (42.5–123.5)	12 (3.5–51.5)	0.001
Barad et al., 2019 [114]	402	36 weeks	Percentage of patients reporting a reduced headache frequency		62%	

## Data Availability

Not applicable.
